# Mental health impacts of climate mitigation and adaptation interventions in high income countries: A scoping review^✰,✰✰^

**DOI:** 10.1016/j.joclim.2026.100728

**Published:** 2026-10-26

**Authors:** Lucy G. Anderson, Frank de Vocht, Sarah Whitmee, Patricia N. Albers

**Affiliations:** a Centre for Public Health, Population Health Sciences, University of Bristol, Bristol, UK; b Centre on Climate Change and Planetary Health, London School of Hygiene and Tropical Medicine, London, UK

**Keywords:** Climate adaptation, Climate mitigation, Public health, Mental health, Planetary health, Health co-benefits

## Abstract

**Introduction:**

Interventions to mitigate and adapt to climate change are increasingly recognized for their potential co-benefits to mental health, although evidence is fragmented. Understanding these co-benefits is important to inform interventions that maximize mental health gains and avoid unintended harms.

**Methods:**

We conducted a scoping review of peer-reviewed primary studies (2015–2025) to map and characterize evidence on the mental health impacts of climate mitigation and adaptation interventions on adults and children in high-income countries. MEDLINE, Web of Science, PsycINFO were searched and study quality was appraised using the Mixed-Methods Appraisal Tool.

**Results:**

Seventeen studies met our inclusion criteria, with most (76%) from the UK or North America. 71% were quantitative quasi-experiments; one was a randomized controlled trial; only two studies were appraised as high quality. Interventions included home retrofits (n = 9), urban greening (n = 3), renewable energy (n = 2), clean air zones (n = 1), diet (n = 1), and behavior change (n = 1). Only one study focused on children. Fourteen (82%) reported at least one improvement in mental health or wellbeing, including all home retrofit studies. Urban greening interventions showed mixed findings, while wind turbines were associated with some negative mental health impacts.

**Conclusion:**

The review suggests climate interventions can offer mental health co-benefits although existing evidence is limited and effects vary by context**.** Considering mental health outcomes in the design and evaluation of climate interventions could enhance equity, effectiveness, and public acceptability. Future research should use consistent outcome measures, disaggregate results by population group, and expand to underexplored sectors and regions.

## 1 Introduction

Climate change is one of the greatest public health challenges of the 21st century [1]. While much scientific and policy attention to date has focused on the physical health impacts of climate change, there is growing recognition of the complex impacts climate change can have on mental health [2]. This is particularly pertinent given that poor mental health is a major public health challenge affecting one in eight people worldwide [3].

The mental health impacts of climate change can arise through multiple pathways. Direct effects include trauma and psychological distress following exposure to climate hazards such as flooding and heatwaves [4]. Indirect impacts can result from the erosion of the social and environmental determinants of mental health such as good quality housing and clean air [1]. Vulnerable groups including children, people with pre-existing health conditions, minority ethnic groups and lower socioeconomic groups are disproportionately affected by the impacts of climate change, exacerbating existing health inequalities [1,4].

Climate mitigation and adaptation interventions are designed primarily to reduce greenhouse gas emissions and adjust to climate hazards, respectively [5]. However they alsomay offer co-benefits or trade-offs to public mental health [6,7]. Understanding these co-benefits is important to guide the design and implementation of interventions to maximize health gains and equity while avoiding unintended harms [7]. Co-benefits may include immediate mental health gains; for example, home insulation can reduce residents’ energy use, easing financial anxiety and improving thermal comfort [8]. Improvements to the social and environmental determinants of mental health, include increased access to greenspace and improving air quality in urban areas, can offer wider population-level benefits [7].

The evidence base around mental health co-benefits is limited and fragmented, partly due to the multi-disciplinary and sometimes siloed nature of climate and health research [7]. Existing reviews have focused on specific interventions such as home retrofits or have concentrated on physical health [9,10]. Evaluations of climate initiatives often prioritize environmental metrics (e.g. emissions reductions or biodiversity gain) over mental health co-benefits, which remain poorly understood, particularly for marginalized groups [7,11,12]. For example, a 2023 scoping review by Dinh et al. [13] explored the health outcomes of a range of climate mitigation policies across transport, housing, and energy but did not include any studies relating to mental health. A 2023 review explored the mental health outcomes of climate adaptation and mitigation interventions but focused on low and middle-income countries only, recognizing their heightened vulnerability to the impacts of climate change [12]. The review identified positive mental health outcomes across a range of intervention types but noted the scarcity of primary studies in this area and low quality of study designs [12].

A clearer understanding of the potential mental health co-benefits and/or unintended consequences of climate interventions would inform the design of equitable climate interventions which maximize mental health co-benefits [11]. Making these impacts visible is critical for identifying and managing trade-offs, capturing the full societal benefits of climate action, and ensuring that mental health outcomes are appropriately reflected in policy appraisal and cost–benefit analyses [14]. It is also critical to identify gaps in the evidence base, so that future climate and mental health research can be effectively prioritized. Our scoping review set out to ask the following research question: What evidence exists about the impact of climate mitigation and adaptation interventions on the mental health and wellbeing of adults and children in high-income countries? In line with the objectives of a scoping review, we sought to map and characterize the available evidence base rather than determine intervention effectiveness. We also sought to explore which types of climate mitigation and adaptation interventions have been studied in relation to mental health; which mental health and wellbeing outcomes are being assessed; how these outcomes have been measured; and how mental health outcomes vary across different population groups (e.g. age, gender, ethnicity, socioeconomic status) for which data are available.

## 2 Methods

### 2.1 Protocol registration

Our protocol followed the Joanna Briggs Institute guidance for scoping reviews [15] and the Preferred Reporting Items for Systematic Reviews Extension for Scoping Reviews (PRISMA-ScR) [16]. Consistent with JBI guidance, the review aimed to map the extent, range and characteristics of the available evidence and identify knowledge gaps. It was registered on Open Science Framework in June 2025 before literature searches commenced (https://osf.io/2pqry).

### 2.2 Definitions

We used the Intergovernmental Panel on Climate Change definition of climate mitigation interventions as *measures designed to reduce greenhouse gas emissions*, while climate adaptation interventions included *initiatives to adapt to climate hazards such as heatwaves and flooding, minimizing harm* [5].

We followed Stuart-Ruano’s definition of mental health and wellbeing by including outcomes related to mental disorders (e.g. depression, anxiety, post-traumatic stress disorder) as well as outcomes related to general psychological wellbeing and quality of life, but excluded neurodegenerative disorders [17].

### 2.3 Search strategy and selection criteria

Three electronic databases were searched for relevant peer-reviewed literature: Web of Science, MEDLINE and PsycINFO. They were selected through consultation with a research specialist librarian to cover both health and non-health specialist literature given the interdisciplinary research question. Our search strategy included climate change terms; terms relating to climate mitigation, climate adaptation, and net zero interventions; and mental health terms (Table S1, Supplementary Materials). Searches were limited to human studies, published in English-language peer-reviewed journals between 2015 and 2025.

In addition to database searches, we hand-searched the reference lists of systematic reviews identified during the searches, and performed backward-forward searches of the reference lists of the included studies and those studies that had cited the included studies.

Search results were indexed in Zotero and imported into Rayyan for duplicate removal and two-stage screening [18]. One reviewer (LA) screened all titles and abstracts while a second reviewer (PA) independently co-screened a randomly generated set of 10% of titles and abstracts. Disagreements (<1% of articles at the title/abstract stage and 2 articles at full-text stage) were resolved through discussion once blinding was removed.

### 2.4 Eligibility criteria

Eligible studies had to involve an intervention or initiative with the primary goal of mitigating or adapting to the impacts of climate change in a country classified as high-income according to the World Bank in 2025 [19]. They also had to report at least one mental health or wellbeing outcome from the population affected by the climate adaptation/mitigation intervention. All primary study designs were included. Subject-relevant systematic reviews and meta-analyses were excluded, but their reference lists were hand-searched. Full inclusion and exclusion criteria are presented in Table 1.

**Table 1:** Overview of the inclusion and exclusion criteria used during the screening process.

	Inclusion criteria	Exclusion criteria
Publication type	Peer-reviewed journal articles published 2025–2015.	Non peer-reviewed studies (e.g. conference papers, books, grey literature).
Population	Adults and/or children.	-
Concept	The study must describe an intervention, activity or initiative with the primary goal of mitigating or adapting to the impacts of climate change (e.g. reducing greenhouse gases directly, or tackling urban heat, air pollution, flooding, improving drought resilience etc.).	Interventions that are designed to improve biodiversity or increase greenspace but that do not make an explicit link to climate change adaptation or mitigation as a goal of the intervention.
Context	Studies conducted in high-income countries, according to the World Bank in 2025.	Studies conducted in low-or middle-income countries, according to The World Bank classification (2025).
Outcomes reported	Studies must report one or more mental health or mental wellbeing outcome(s) from the population affected by the climate adaptation or mitigation activity or intervention.	Physical health outcomes only.
Study design	All types of primary study design that report health outcomes will be included (e.g. experimental or quasi-experimental trials, case studies, evaluations, health impact assessments, pilot studies etc.)	Secondary studies.
Language	English language articles.	Non-English language articles.

### 2.5 Data extraction and charting

Key characteristics of each study were extracted from the full texts into a data charting template in Microsoft Excel. The categories were agreed by the research team and the form was piloted by LA and PA. One reviewer (LA) extracted the data, but uncertainties around specific papers were discussed with a second reviewer (PA) . Charting categories included details of the publication (author, year, publication), intervention type (e.g. home retrofit, urban greenspace creation), climate hazard being addressed (e.g. urban heat, flooding), study setting (locality, country), study period (dates, duration), mental health outcome measures used, key results, sample population size and characteristics (where provided).

### 2.6 Synthesis of results

Data were synthesised narratively due to the heterogeneity of intervention types, study designs, populations, and mental health outcome measures. Studies were grouped by intervention type and summarized descriptively, including intervention characteristics, study design, population, mental health outcomes assessed, and key findings. The synthesis focused on mapping the extent, nature, and characteristics of the available evidence, identifying patterns across studies, and highlighting evidence gaps rather than assessing intervention effectiveness.

### 2.7 Quality assessment

To appraise the quality of the included studies, we used the Mixed Methods Appraisal Tool (MMAT) which allows the use of a consistent tool across diverse study designs, including randomized controlled trials (RCTs), quasi-experimental, cross-sectional, qualitative, and mixed methods studies [20]. Each study was rated against five criteria relating to its specific design. We summarized each study’s quality using a five-star system based on the number of criteria met (e.g. ***** for 5/5), as recommended within the MMAT guidance [21]. Study quality results were used for transparency only. No studies were removed from the review based on their quality.

## 3 Results

### 3.1 Study selection

In total, 1,574 studies were identified through database searches and hand-searching relevant articles in January 2026. After removing 333 duplicates, 1,241 titles and abstracts were screened against the eligibility criteria. Of these, 60 full texts were assessed for eligibility, and 17 articles met the criteria and were included in the review (Fig. 1).

**Fig. 1:**
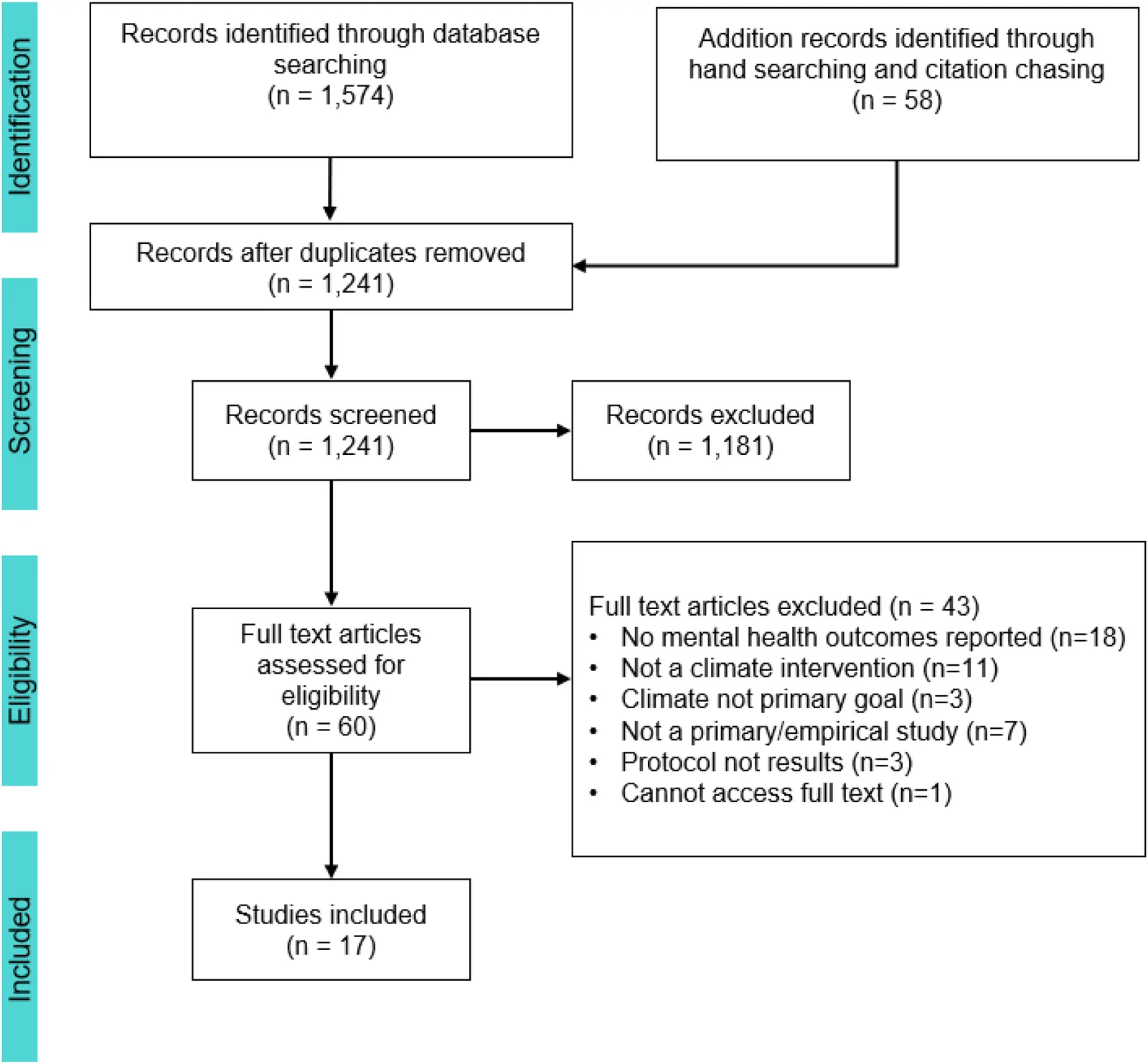
PRISMA flow diagram of study selection process.

### 3.2 Study characteristics

The 17 studies varied greatly in terms of the interventions they explored, their duration, study design and outcomes measured (Table 3). The studies collected data between 2005 and 2021 with study time frames ranging from a one-time cross-sectional survey of park visitors [22], to longitudinal studies lasting five years or more [[23], [24], [25], [26]]. Six studies (38%) were conducted in the UK [[23], [24], [25], [26], [27], [28]], four (25%) in the USA [[29], [30], [31], [32]], two (12.5%) in Canada [33,34], and one in each of Spain [35], Italy [22], Germany [36], Ireland [37] and Australia [8] (Fig. 2). Sample sizes ranged from 16 to 10,009 participants [23,29]. Two modeling studies (one on home retrofits in the UK [38], one on an urban greening initiative in Barcelona, Spain [39]) met our inclusion criteria but were excluded after discussion between reviewers as they were projection models rather than empirical studies.

**Fig. 2:**
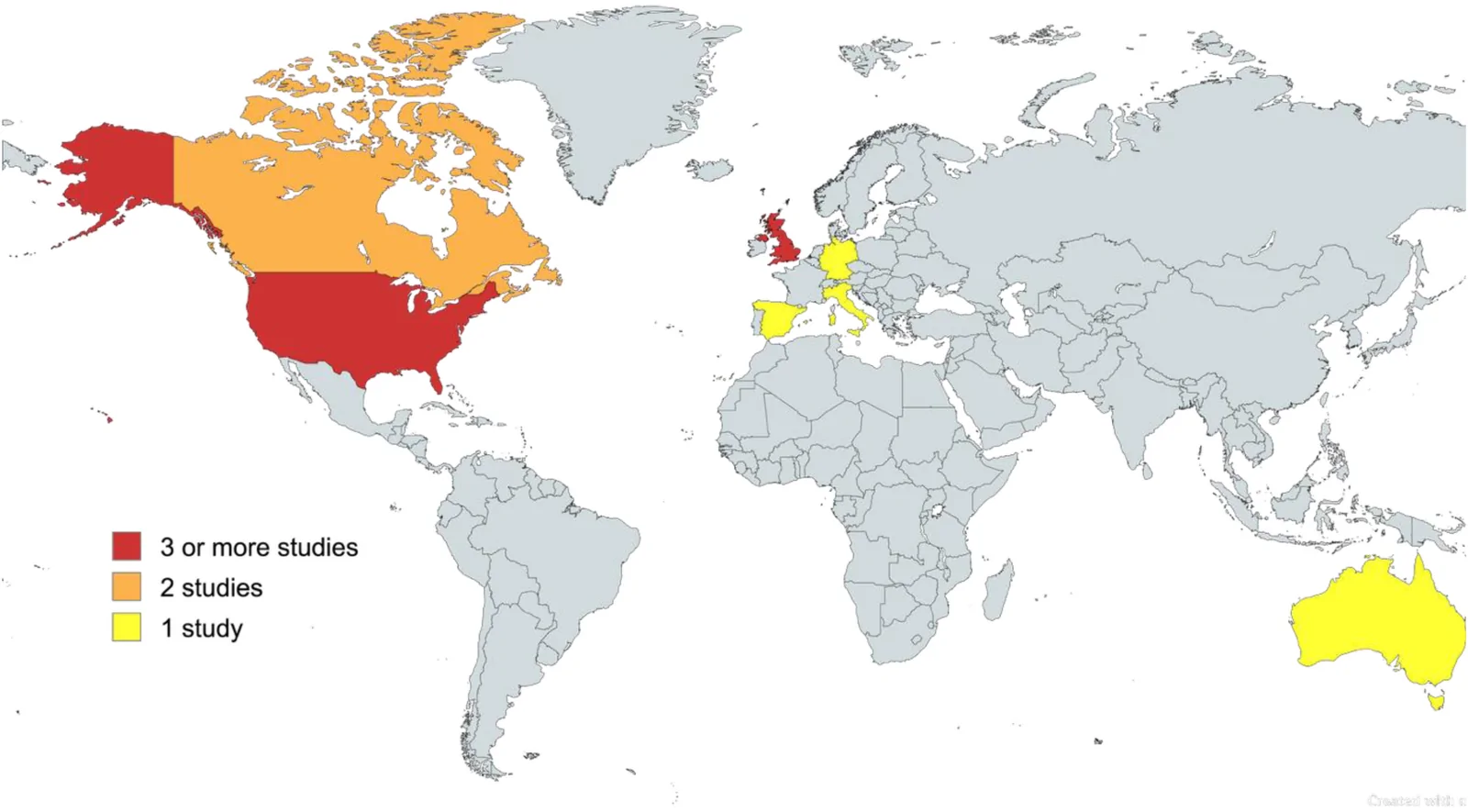
World map showing the geographic distribution of the 17 included studies.

### 3.3 Study designs

A wide range of study designs were included (Table 3) including only one RCT [8]. Of the remainder, twelve were pre-post quasi-experimental evaluation studies of which seven included a control group [[25], [26], [27],^31^,^32^,^35^,^36^] and five did not [23,28,30,33,37]. Other designs included a quantitative difference-in-difference analysis which combined air quality and mental health data [24], a cross-sectional study [22], a mixed-methods feasibility study [29] and a mixed-methods lived experience study [34] (Table 3).

### 3.4 Types of climate intervention studied

Around half of the 17 studies (53%) explored the mental health outcomes of retrofit interventions to improve home insulation and energy efficiency [8,23,25,27,28,[30], [31], [32],^37^]; three studies (22%) explored green urban infrastructure to tackle the impacts of urban heat or prevent flooding [22,26,35]. Two studies explored the impact of new wind turbine developments for renewable energy (11%) [33,34]. Three studies explored other interventions including the impacts of low emission zones [24]; a climate behavior change course designed to motivate individual behavior change relating to diet, travel and energy use [29]; and the introduction of the Lancet’s Planetary Health (low-carbon) diet in a hospital canteen [36] (Table 3).

### 3.5 Mental health outcomes assessed

A variety of mental health outcomes were measured across the 17 included studies. Twelve studies (71%) used validated mental health scales, most commonly the mental component scores derived from Health-Related Quality of Life (HRQoL) survey instruments which are designed to assess overall mental functioning and wellbeing (Table 2). They included the 36-Item Short Form Survey (SF-36; 4 studies), and 12-item Short Form Survey (SF-12; 3 studies), the EuroQol 5 Dimension survey (EQ-5D; 1 study), and KIDSCREEN-27 (1 study), a HRQoL tool adapted to assess children and adolescents (Table 3). The Warwick-Edinburgh Mental Wellbeing Scale (WEMWBS), a questionnaire measuring positive mental wellbeing, was also used by three studies (Table 3). Of the remaining studies, two used indicators from national or regional routine surveys [24,30], and two developed custom surveys [23,34].

**Table 2:** Overview of the validated mental health measures referenced in the study results.

Survey acronym	Full name	What it measures	What the result means
SF-36	36-Item Short Form Survey [42]	Generic health related quality of life (HRQoL) instrument with Mental Component Summary (MCS) covering vitality, social functioning, role-emotional and mental health domains.	Higher score = better mental health/functioning.
SF-12	12-item Short Form Health Survey [43]	Abbreviated version of the SF-36 producing Mental Component Summary score reflecting psychological distress and wellbeing.	Higher score = better mental health.
EQ-5D	EuroQol 5 Dimensions Questionnaire [44]	HRQoL tool including an anxiety/depression dimension and overall health index.	Higher index score = better health; For the anxiety/depression item, a lower score = fewer problems.
KIDSCREEN-27	KIDSCREEN-27 Health-Related Quality of Life Questionnaire [45]	Multidimensional HRQoL measure for children/adolescents including psychological wellbeing domain.	Higher scores = better wellbeing.
WEMWBS	Warwick-Edinburgh Mental Wellbeing Scale [46]	Measure of positive mental wellbeing (optimism, usefulness, relaxation, relationships).	Higher scores = greater positive wellbeing.
WHO-5	World Health Organization Five Wellbeing Index [47]	Self-report instrument measuring wellbeing using five statements relating to the past 2 weeks.	Higher score = better mental wellbeing.

**Table 3:** Characteristics of the included studies. QS = quality appraisal score using the MMAT.

Author, year	Country	Climate hazard	Intervention	Study duration	Study design	Mental health outcomes used	Main result	Sample size and population	QS *
Ahrentzen et al. (2015) [30]	Arizona, USA	Extremes of heat and cold	Home retrofit.	2010–2012	Quantitative, repeated measures, no control.	Questionnaire items on mental health drawn from two annual surveys: The US National Health Interview Survey and The Behavioral Risk Factor Surveillance System for Arizona.	• Improved insulation and a reduction in temperature peaks led to reduced emotional distress (fixed effects regression: t = −2.085, p < 0.05) and increased quality of life/health (fixed effects regression: t = 3.179, p < 0.01) among participants, one year after the intervention.	N = 57 (no control). Older adults, low-income, mean age 73 (range 62–93). 74% female, 88% lived alone.	***
Beshir & Fichera (2025) [24]	London, UK	Air pollution	Clean Air Zone	Baseline data 2005–2009, follow up data 2016–2019	Quantitative difference-in-difference analysis compared exposed areas in Greater and Central London to comparable unexposed areas in England before/after introduction of Ultra Low Emission Zone (ULEZ).	Wellbeing data from the UK Office of National Statistics Annual Population Survey. Mental wellbeing was measured using Likert scales (0 = not at all, 10 = completely): How satisfied they are with life nowadays; to what extent they feel that the things they do in life are worthwhile; how happy they felt yesterday; how anxious they felt yesterday.	• Increased feelings of happiness (+1.8%), worthiness (+1.9%) and satisfaction (+2.1%) post-ULEZ compared to control regions. • Anxiety also reduced by 11% among individuals working in Central London post-ULEZ compared to control regions.	Modelled from representative national survey data from c.98,000 adults aged 16–64 2016 (baseline) and 2019 (post-intervention).	****
Breysse et al. (2025) [31]	Minnesota, USA	Extremes of heat and cold	Home retrofit	2010–2013	Quantitative, repeated measures design with intervention and control groups.	Mental component score (MCS) from VR-12 (Veterans RAND 12-Item health survey, adapted from SF-36). Mean number of good mental health days in past month.	One year post-intervention: • Increased MCS (+1.6 in intervention group vs. −1.5 in control group, P=.026). • Higher No good mental health days in the past month (+1.9 in intervention group, vs −2.8 in control group, P = 0.23).	N = 80 (n = 40 intervention, n = 40 control). Intervention group comprised 95% non-Hispanic white, 70% female, median age 66, low income. Control group was age and ethnicity matched low-income Minnesota residents from an existing regional survey.	****
Curl et al. (2015) [25]	Glasgow, UK	Extremes of heat/cold	Home retrofit	2006 - 2011	Quantitative repeated cross-sectional study with a nested longitudinal cohort.	Mental component score derived from SF-12	Fabric works (warmth interventions including loft and cavity wall insulation) had positive impacts on resident mental health 1–2 years after intervention (MCS +1.84, 95% CI 0.04 to 3.65).	1933 (n = 1334 intervention, n = 602 control). Deprived communities. Specific demographics of sample not provided.	***
Grabow et al. (2018) [29]	Madison, WI, USA	Climate mitigation	Individual behavior change course	2017	Mixed method feasibility study. Repeated measures, no control.	Five survey instruments were tested as part of the feasibility study: Center for Epidemiologic Studies Depression Scale (CES-D); Pemberton Happiness Index (PHI); Stanford Presenteeism Scale (SPS-6); Perceived Stress Scale (PSS-10); MCS score from SF-36.	After completing the course, results from all instruments slightly improved, reducing slightly for CES-D and PSS-10 which measure risk of depression and perceived stress, and increasing slightly for SPS-6, PHI, and MCS which measure presenteeism, happiness and mental health. For example, the mean MCS increased from 65.6 (baseline) to 69.5 (six months post-intervention). • No statistical analyses were conducted due to small sample/feasibility study.	N = 16 participants, mean age 39.7 (range 30–63), 7 college graduates, 9 college post-graduates. (no control group)	***
Grey et al. (2017) [27]	Wales, UK	Extremes of heat and cold	Home retrofit	2013 - 2015	Quasi-experimental pre-post intervention and control group.	MCS score from SF-12. Subjective wellbeing (using ONS questions).	• Subjective wellbeing increased in the intervention group post-retrofit (B = 0.38, 95% CI 0.12 to 0.65). • No change in MCS post-retrofit.	782 (n = 364 intervention, n = 418 control). Residents of low-income neighbourhoods, 79% household income <£29,999. 58% female: 41.2% male, 25% received housing benefit.	****
Harrison et al. (2025) [36]	Heidelberg, Germany	Climate mitigation	Low carbon dietary intervention. (Introduction of the Lancet’s Planetary Health diet in main hospital cafeteria)	2023 (3 months)	Quasi-experimental pre- and post-study with an intervention and control group.	Warwick-Edinburgh Mental Wellbeing Scale and HRQoL using SF-36	• Mean WEMWBS increased marginally by +0.86 in the intervention group vs. +0.29 in the control group, leading to a difference-in-differences of 0.61 (95% CI: − 0.78, 1.99; p = 0.39, Cohens d = 0.11). • No significant intervention effects on HRQoL among SF-36 outcomes.	Regular customers (> one visit/week) in the largest cafeteria = intervention group (n = 121) and in all other cafeterias = control group (n = 128). Majority were hospital staff. No sig diff in gender, age, income between control and intervention groups.	***
Hunter et al. (2021) [26]	Belfast, N.Ireland, UK	Flood mitigation/urban heat	Urban greenspace (Creation of urban greenway with flood mitigation element)	2010–11 (baseline) to 2016–17 (follow up)	Repeated cross-sectional study. Treatment and control group	Warwick-Edinburgh Mental Wellbeing Scale	• No intervention effect overall. • Mean WEMWBS increased slightly in the intervention group (from 50.6 pre-intervention to 51.2 post, p = 0.52) compared to a slight decline in WEMWBS in the control group (52.2 to 51.3, p = 0.018).	1214 (968 intervention, 246 control). Intervention group lived ≤ 1 mile from the greenway.44.5% male, mean age 51.7 (SD 19.1). 23.3% lived in most deprived area (1st quintile of N Ireland Multiple Deprivation Measure).	****
Hutchinson et al. (2025) [37]	Dublin, Ireland	Flood mitigation	Home retrofit		Quasi-experimental pre-post evaluation with no control group.	EQ-5D anxiety and depression score; HRQoL from SF-36	• Significant reduction in anxiety and depression score (EQ-5D) mean difference (95% CI) −0.35 (−0.22, −0.49)* (n = 251) • Significant increase in emotional wellbeing (SF-36) mean difference (95% CI): 0.10 (0.09, 0.12)* (n = 511)	N = 546 low income adults. Mean age 68.9 years, 66.2% female.	***
Jalali et al. (2016) [33]	Ontario, Canada	Climate change mitigation (renewable energy transition)	Wind turbines	2014–2015	Quantitative prospective cohort study. Pre-post intervention. No control.	MCS score from SF-12			
Satisfaction With Life Scale (SWLS)	• Significant reduction in MCS after exposure to wind turbines (mean score dropped from 56.07 to 48.24, p = 0.002) with the % of participants classified as “at risk for depression” (MCS ≤ 42) increasing from 0% to 22.6% post-exposure (p = 0.004). • Reduced SWLS score following exposure (from 28.43 to 22.97, p < 0.001) with the % of participants classified as “dissatisfied” (SWLS ≤ 20) rose from 9.7% to 35.5% (p = 0.012). • Participants who reported negative attitudes toward turbines, concern about property devaluation, or visual/noise annoyance experienced the largest declines across all measures.	31 participants (no control). 58% female, 97% homeowners, 80% university educated. Mean age 53.67 (range 30–72).	***						
Page et al. (2025) [8]	Victoria, Australia	Extremes of heat and cold	Home retrofit	2018–2020	Randomised Control Trial	MCS score using SF-36			
	The intervention group experienced a significant increase in MCS score (vs control) after the intervention. (Linear regression: coef = 1.73, 95% CI: 0.21 to 3.25)	984 (488 intervention, 276 control). Mean age 75, low-income households.	*****						
Panno et al. (2017) [22]	Milan, Italy	Urban heat	Urban greenspace (visits to urban park)	September 2015	Cross-sectional survey of park visitors.	WHO-Five Well-Being Index (WHO-5)	Higher frequency of visits to Parc Nord in Milan in summer heat associated with higher mental wellbeing and a lower level of ego depletion.	115 (no control). Mean age 43.27 (SD 16.96). Half (49.6%) had university degree. 50.4% Male: 48.7% Female.	**
Poortinga et al. (2017) [23]	Carmarthenshire, Wales, UK	Extremes of heat/cold	Home retrofit	2009 - 2016	Five-wave repeated cross-sectional study.	Authors created a custom mental health scale, combining five items (e.g. nervousness, sadness, calmness, happiness), scored on a 0–100 scale.	Mental health increased among participants who received loft insulation (B = 4.281 (SE =0.660) p < 0.001) and external wall insulation (B = 2.657 (SE=0.823) p < 0.01). However, the installation of cavity wall insulation was associated with poorer mental health (B =−2.052 (SE 0.687) p < 0.05) compared to pre-intervention.	10,009 respondents. Majority 65+, low income and recipients of housing benefit.	****
Sanz-Mas et al. (2025) [35]	Barcelona, Spain	Extremes of heat/cold	Urban greenspace (School playground green/blue/grey space)	2020–2021	Quasi-experimental pre-post mixed-methods evaluation with control and intervention schools.	KIDSCREEN-10 index from KIDSCREEN-27 validated survey tool.	Boys in the intervention schools had improved psychological wellbeing (β = 1.87, 95% CI=−0.17 to 3.91) post-intervention. They also had a significant increase in HRQoL (β = 3.09, 95%CI=1.14 to 5.04). No change in psychological wellbeing (β −0.04, 95% CI −2.29 to 2.22) or quality of life (β −0.04, 95% CI − 1.49 to 2.30) between intervention and control groups for girls of the same age. Qualitative insights included greater feelings of relaxation, safety and physical comfort in the schoolyard post-intervention. Gender differences were attributed to differences in play behaviour.	792 (407 intervention, 385 control). Mean age 11.3 (0.5), 44.7% female: 55.3% male.	*****
Sawyer et al. (2022) [28]	East Sussex, UK	Extremes of heat/cold	Home retrofit	2016 - 2018	Mixed methods pre-post evaluation study. No control.	Warwick-Edinburgh Mental Wellbeing Scale	WEMWS scores increased post-intervention (Mean = 42.49, SD = 9.83) compared to pre-installation (M = 39.31, SD = 11.06). This difference was significant, t(77) = 3.42, p = .001. Qualitative insights also showed that people living with depression said having a working heating system and being warm helped with their mental health, as did a sense of control over the temperature (having a thermostat vs. night storage heating).	78 (no control). Mean age 60.5 (range 22 to 93), 71.4% female, 97% white British, 57.7% unemployed, 34.6% retired. N = 23 in interview group (similar characteristics).	***
Tonn et al. (2018) [32]	USA-wide	Extremes of heat/cold	Home retrofit	2008–2010	Quasi-experimental pre-post study with intervention and wait-list control groups.	No. days of poor mental health in the past month	No. of days of poor mental health dropped from 7.1 to 3.7 days/month post-retrofit (a 48% reduction, p<.001) one year post-retrofit.	1468 (665 intervention, 803 wait-list controls) participants randomly selected from national programme to participate in the analysis.	***
Walker et al. (2015) [34]	Ontario, Canada	Climate change mitigation (renewable energy transition)	Wind turbines	2011–2012	Mixed methods lived experience study (one time point). Guided by grounded theory.	Interview questions relating to impacts of turbines on quality of life. Survey questions designed to explore interview themes. No validated mental health tools used in survey.	14/16 interviewees mentioned the impact that wind turbines had impacted their quality of life in some way in the 3 years since they were implemented. They had ranged from a minor annoyance to "ruining their existence" and the noise causing sleep disruption. Impacts were greater for those who opposed the wind turbines and intra community conflict (between supporters and opponents) exacerbated the issues. Despite this 72% supported the wind turbines.	26 interviewed living within 1 km of turbines comprising 38% female, homeowners aged 40+ years.152 completed survey, including residents within 2 km of wind turbines with similar demographics to interviewees.	***

### 3.6 Quality of studies

The results of the quality appraisal are presented in Table 3 with further details in Table S2 (supplementary materials). Sixteen of the 17 studies met at least three of the five criteria according to the MMAT, but only two were appraised as high quality (5/5 criteria met), including the only Randomized Control Trial (RCT) study and a quasi-experimental study of school playground climate interventions [8,35].

### 3.7 Key findings

Fourteen studies (82%) reported improvements in mental health or wellbeing following the intervention. The nine studies on home energy retrofits consistently found positive effects on at least one mental health-related outcome (Table 3). For example, Tonn et al. [32] reported a 48% reduction in poor mental health days post-retrofit (from 7.1 to 3.7 days per month); Sawyer et al. [28] reported an increase in WEMWBS scores from 39.3 to 42.5 (p = 0.001) and Poortinga et al. [23] reported improvements in mental health (custom scale) for loft insulation (+4.28 points on a custom 1–100 mental health scale, SE = 0.66, p < 0.001) and external wall insulation (+2.66 points, SE= 0.82, p < 0.01), respectively (Table 3). Other studies reported increases in mental component scores (SF-12/SF-36 + 1.6 to +1.84; [8,25,31]) and improvements in quality of life ([8,25,31]) or reduced anxiety [8,30,31]. However, Grey et al. [27] found no change in mental component scores after retrofit, though subjective wellbeing increased. Cavity wall insulation without adequate ventilation was associated with a decline in mental health score (−2.05 points on a 0–100 scale; SE = 0.687, p < 0.05) in a large scale home retrofit program in Wales [23].

The three studies exploring urban greening interventions showed more mixed results (Table 3). A schoolyard heat adaptation intervention led to improved psychological wellbeing and quality of life among boys but not girls in Barcelona primary schools [35] (Table 3). The creation of an urban greenway in Belfast which doubled as a flood prevention measure only led to marginal improvements in mental wellbeing among local residents (mean WEMWBS +0.6, p = 0.52) [26]. While a low-quality cross-sectional study of park visitors in Milan showed lower ego-depletion (β=−0.55, 95% CI −0.98 to −0.12) and higher mental wellbeing (+0.43 points on the WHO-5 scale, 95% CI −.38 to 1.24) among those who visited the city’s large Parco Nord Milano more frequently during summer [22]. However, the study had no control group, response rate, or consideration of other greenspace visits.

The two studies on wind turbines reported declines in mental health and quality of life among residents living near turbines with a mean drop in SF-12 MCS from 56.1 to 48.2 (p = 0.002) and reduced life satisfaction with up to 22.6% of participants classified as at-risk for depression post-exposure, especially among those with negative attitudes or higher noise sensitivity [33,34].

Of the three other studies, the implementation of the London Low Emission Zone led to increased feelings of happiness (+1.8%), worth (+1.9%) and life satisfaction (+2.1%) and reduced anxiety by 11% [24], while a climate education course [29], and the introduction of low-carbon food options in a hospital canteen [36] led to marginal improvements in mental wellbeing (Table 3).

### 3.8 Outcomes for different population groups

Few studies disaggregated mental health outcomes by demographic characteristics. Most of the home retrofit studies focused specifically on low-income populations with five focusing on older adults as they were the target for fuel poverty interventions [8,23,28,30,31]. Only one study by Sans-Maz et al. [35] focused specifically on exploring the mental health outcomes in children. The study, which explored the effect of a school-based climate adaptation intervention reported that the intervention had led to improvements in psychological wellbeing (β = +1.87, 95% CI −0.17 to 3.91) and quality of life (β = 3.09, 95%CI=1.14 to 5.04) among 11–12 year old boys but found no change in psychological wellbeing (β = −0.04, 95% CI −2.29 to 2.22) or quality of life (β −0.04, 95% CI − 1.49 to 2.30) among girls of the same age (Table 3), a result that was attributed to differences in their play-time behaviours [35].

## 4 Discussion

This scoping review identified 17 studies exploring the mental health and wellbeing impacts of climate mitigation and adaptation interventions in high-income countries. The review mapped a small but growing and highly heterogeneous evidence base spanning multiple intervention types, study design and outcomes measures. Although interventions, study designs, timeframes, and outcomes varied widely, most (14 of 17) studies reported improvements in at least one mental health-related outcome measure suggesting that climate interventions may offer promising co-benefits to public mental health. However, these findings should be interpreted as a description of the current evidence landscape rather than evidence of intervention effectiveness.The strongest and most consistent evidence came from home energy retrofit interventions, where all nine studies reported meaningful improvements in mental wellbeing, while mental health outcomes from green infrastructure interventions were more mixed [22,26,35]. The two studies focusing on wind turbines revealed the potential for unintended psychological stress [33,34]. However, these studies should be treated cautiously as they were designed explicitly to investigate potential adverse health effects of wind turbines. Importantly, they highlighted the importance of community perceptions, noise sensitivity and local acceptability in shaping mental health outcomes [33,34].

Evidence on mental health outcomes was very limited or missing for a number of key areas of climate mitigation and adaptation, as summarized in Fig. 3. Only one study featured a greenspace that also served as a flood adaptation measure [26] and only one study evaluated a clean air intervention [24] despite flooding and air pollution posing major health threats to high-income countries. Moreover, we did not identify studies exploring the impact of water security, decarbonizing the healthcare sector, or public/active transport strategies on mental health despite these being recognized priorities in the UK and elsewhere (Fig. 3).

**Fig. 3:**
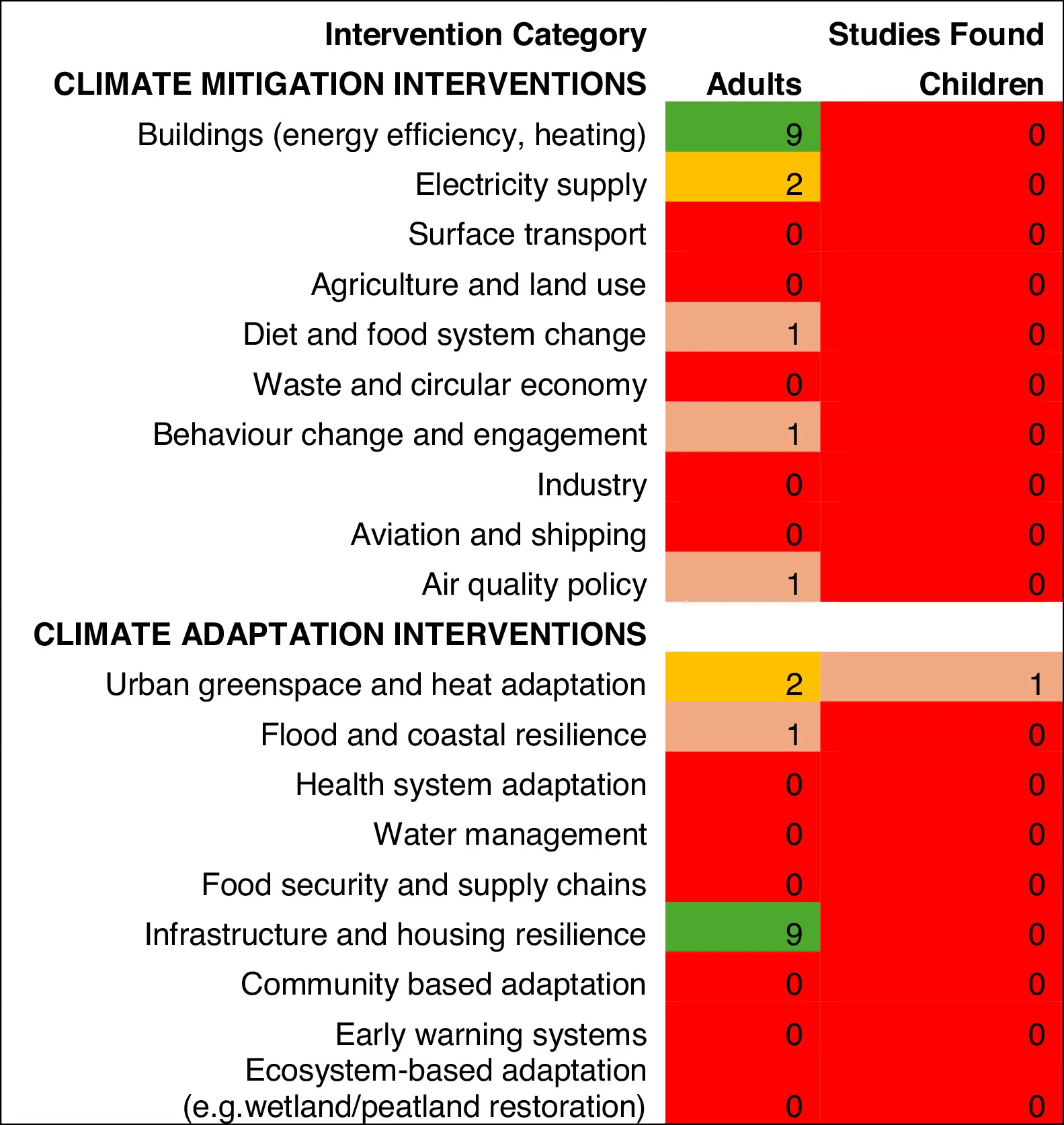
Evidence gap map of studies reporting mental health outcomes related to climate mitigation and adaptation interventions in high-income countries. Interventions are categorized according to IPCC and the UK National Adaptation Programme frameworks. The number of studies identified for adults and children is shown for each intervention type.

There were also gaps in population focus, with only one study focusing on children’s mental health identified, despite their recognised vulnerability to climate risks [40]. Most retrofit studies focused on low-income households and often older adults which helps to assess equity among that group but not beyond it. This represents an important evidence gap given concerns that both climate change and poor mental health disproportionately affect vulnerable populations. Without more consistent subgroup analyses, it remains unclear whether climate interventions reduce, maintain, or exacerbate existing mental health inequalities. Ten of the seventeen studies had been conducted in the UK and USA with many European countries, New Zealand and East Asia seemingly not yet evaluating mental health outcomes of climate interventions. This geographic concentration limits understanding of how mental health outcomes may vary across different policy, housing, cultural and climatic contexts.

Compared with Flores et al. [12] systematic review of climate interventions in low- and middle-income countries (LMICs), we found differences in both interventions and methodological quality. LMIC studies understandably tended to focus on acute climate adaptation strategies (e.g. flood resilience, and water/food security) whereas studies in this review primarily explored mitigation strategies such as home energy retrofits to reduce fuel use. A greater proportion of the high-income country studies in this review used validated mental health measures (71%) than in the LMIC review (13%) [12]. However, most included studies in both LMIC and high income contexts reported positive mental health co-benefits of climate interventions.

Within this study, differences in study design, outcome measurement, and follow-up periods made synthesis across studies difficult and may hinder a meta-analysis at present. For example, follow-up surveys ranged from three weeks to several years post-intervention, and some studies lacked a control group, or used mental health measures that could not be directly compared. Disaggregation of results by gender, age, or socioeconomic status was also missing, making it difficult to understand the impact of climate interventions on vulnerable groups. Where subgroup analysis occurred in a study of green, blue, and grey climate cooling infrastructure in Barcelona schoolyards, the intervention was shown to improve wellbeing among boys but not girls, suggesting differing impacts across population groups [35]. Future evaluation designs of interventions should include sub-group analyses to explore this further.

Home retrofit interventions were particularly diverse in their design and delivery and included different types of retrofits within them. For example, some only focused on thermal insulation [8] while others included kitchen and bathroom upgrades [25] making broad comparisons challenging. While most studies reported improvements in mental health outcomes following retrofits, such benefits are not guaranteed. For example, Poortinga et al. [23] found that while loft and external wall insulation (installed alongside extractor fans) were associated with improved mental wellbeing, cavity wall insulation installed without ventilation was linked to poorer mental health outcomes, reportedly because of increased humidity exacerbating respiratory health conditions. This highlights the importance of carefully monitoring the differing designs and health impacts of such interventions.

### 4.1 Limitations

This scoping review has several strengths. To our knowledge, it is the first to synthesize empirical evidence on mental health and wellbeing outcomes of climate mitigation and adaptation interventions in high-income countries, addressing an important gap in the literature that has previously focused on physical health outcomes, modeling studies, or mental health and wellbeing in LMIC settings. The review followed established best practice, with a prospectively registered protocol and reporting guided by JBI and PRISMA-ScR frameworks [15,16]. The interdisciplinary search strategy was developed with a specialist research librarian and spanned health and non-health databases, reflecting the cross-sectoral nature of climate and health research. The inclusion of a structured quality appraisal using the MMAT also supported transparent interpretation of the heterogeneous evidence base and helped to identify key gaps for future research.

However, our review has several limitations. Only three databases were screened for literature and we only had resources to co-screen 10% of titles and abstracts. Data extraction was conducted by a single reviewer, and although uncertainties were discussed with a second reviewer, this could have introduced reviewer bias. Similarly, while the MMAT allowed us to consistently appraise a wide range of study types, only one reviewer conducted the quality appraisals which also could have introduced bias.

As a scoping review, the purpose of this study was to map and characterize the available evidence on mental health outcomes associated with climate mitigation and adaptation interventions, rather than to determine intervention effectiveness. Nevertheless, several limitations of both the review methods and the underlying evidence base should be considered when interpreting the findings. Attributing observed changes in mental health and wellbeing directly to climate mitigation and adaptation interventions is challenging. Many included studies used observational, quasi-experimental, cross-sectional, or uncontrolled pre-post designs which, while often appropriate for evaluating complex real-world interventions, do not permit strong causal inference. The extent to which individual studies accounted for potential confounding factors also varied, raising the possibility of residual confounding. Furthermore, relatively few studies explored the specific mechanisms through which interventions may have influenced mental health outcomes, making it difficult to disentangle intervention effects from wider social, economic, environmental, or policy influences in some cases.

Excluding grey literature from the search could also have omitted relevant local authority or non-governmental organization (NGO) evaluations. The evidence base was also geographically concentrated in a small number of Western high-income countries, particularly the UK and USA. No studies were identified from several high-income regions, including East Asia. It is unclear whether this reflects a true evidence gap, differences in evaluation and reporting practices, or the restriction to English-language peer-reviewed literature. Finally, as this is a relatively nascent area of research, a diverse range of terms are used to describe climate interventions, meaning that although our search strategy was created from key policy documents and reviewed by subject matter experts, some interventions may have been missed from the search.

### 4.2 Implications for policy and research

Recognizing the potential mental health co-benefits of climate interventions could strengthen public and political support for ambitious climate action. Framing contentious policies like low emission zones in terms of public mental health gains rather than simply environmental stewardship could help to shift narratives away from cost and inconvenience and towards health equity and improved quality of life, reducing public resistance. Integrating mental health outcomes into Net Zero strategies also could improve the equity and effectiveness of climate responses [41]. Several study protocols that aimed to evaluate both mental health and environmental outcomes of air quality interventions were discovered during screening suggesting that momentum may be building in this area. However, more evidence is needed to assess co-benefits and trade-offs in a systematic way [7,11].

### 4.3 Conclusion

This scoping review shows the potential for climate interventions to offer important mental health co-benefits in high-income countries; however evidence remains limited at present. Future studies should integrate mental health outcomes into the evaluation of a wider range of climate mitigation and adaptation interventions to fill the evidence gaps this study has highlighted. This should include the use of consistent, validated mental health measures to facilitate future meta-analysis and the collection of disaggregated data to identify which groups benefit, as well as any that may be left behind. A stronger evidence base has the potential to help decision makers maximize population mental health and equity and improve public acceptability for climate action, while avoiding unintended harms. This should help to ensure that climate strategies not only meet Net Zero objectives but simultaneously contribute to healthier, more equitable societies.

## Funding sources

LA is funded by the National Institute for Health and Care Research (NIHR) School for Public Health Research (SPHR), Grant Reference Number NIHR204000. The views expressed are those of the author(s) and not necessarily those of the NIHR or the Department of Health and Social Care.

## CRediT authorship contribution statement

**Lucy G. Anderson:** Writing – review & editing, Supervision, Methodology, Data curation, Conceptualization. **Frank de Vocht:** Writing – review & editing, Supervision, Methodology, Conceptualization. **Sarah Whitmee:** Writing – review & editing, Supervision, Methodology, Conceptualization. **Patricia N. Albers:** Writing – review & editing, Supervision, Methodology, Data curation, Conceptualization.

## Declaration of competing interest

The authors declare that they have no known competing financial interests or personal relationships that could have appeared to influence the work reported in this paper.

## Footnotes

Supplementary material associated with this article can be found, in the online version, at doi:10.1016/j.joclim.2026.100728.
