# Supplementary material for: Mental health impacts of climate mitigation and adaptation interventions in high income countries: A scoping review✰,✰✰
Source: J Clim Chang Health. 2026 Oct 26;32:100728. doi: 10.1016/j.joclim.2026.100728 (PMC13734810; doi:10.1016/j.joclim.2026.100728)
Supplement: Supplementary file 1 [file mmc1.docx]

**Supplementary Materials**

| **Table S1.** Specific search terms for OVID Medline database. The search string was formatted to the specific syntax of Web of Science and PsycINFO but the terms remained the same. | | |
| --- | --- | --- |
| Climate change terms | 1 | exp climate change/ |
|  | 2 | global warming/ |
|  | 3 | greenhouse gas/ |
|  | 4 | carbon footprint/ |
|  | 5 | exp extreme weather/ |
|  | 6 | exp air pollution/ |
|  | 7 | exp climatic processes/ |
|  | 8 | drought/ |
|  | 9 | (climat* adj3 (chang* or varia* or disrupt* or hazard* or impact* or vulnerab* or function* or extreme or catastroph* or emergenc* or shift* or dynamic* or sensitiv* or footprint)).ti,ab,kw. |
|  | 10 | (global or planet* or world or climate) adj1 (heating or warming).ti,ab,kw |
|  | 11 | ((temperature* or cold or heat or hot) adj4 (extreme or elevate* or irregular or intense or reduced or pattern* or global or stress)).ti,ab,kw. |
|  | 12 | (heatwave or heat-wave or thermal stress or thermal comfort or “urban heat island”).ti,ab,kw. |
|  | 13 | (Coldwave* or cold-wave* or “cold wave”).ti,ab,kw |
|  | 14 | ((sea-level or sea level) adj5 (ris* or chang*)).ti,ab,kw. |
|  | 15 | (storm* or hurricane* or cyclone* or typhoon* or windstorm* or "extreme wind" or “tidal surge”).ti,ab,kw. |
|  | 16 | flood*.ti,ab,kw. |
|  | 17 | ((rain* or precipitation) adj3 (extreme or elevat* or irregular or intense or reduc* or pattern*)).ti,ab,kw. |
|  | 18 | (Wildfire or "wild fire" or wild-fire or bush fire or bush-fire or bushfire).ti,ab,kw. |
|  | 19 | or/1-18 |
| Climate mitigation/  adaptation intervention terms | 20 | ("climate mitigation" OR “climate adaptation” OR "net-zero" OR “net zero” OR decarbonis* OR decarboniz*OR "carbon sequestration" OR “climate resilience” OR "co-benefit" OR “climate action*” OR “circular economy” OR (carbon adj3 (“capture" OR "cut" OR low* OR "limit" OR "reduce")) OR "just transition" OR “energy transition” OR “nature-based solution” OR “climate solution” ).ti,ab,kw. |
|  | 21 | ("energy efficiency" OR “clean energy” OR "building insulation" OR "passive cooling" OR "cool roofs" OR "green roofs" OR "street tree*" OR "urban tree*" OR "flood defence*" OR "urban drainage system*" OR "climate-resilient infrastructure" OR "urban greening" OR "green street*" OR "green infrastructure" OR "green roof" OR "urban greenspace" OR “urban heat reduction” OR "nature-based solution*" OR "afforestation" OR “forest protection” OR "peatland restoration" OR "wetland restoration" OR "coastal habitat restoration" OR "water conservation" OR "water resilience" OR "rainwater harvesting" OR "drought-resistant crop*" OR “sustainable agriculture” OR “sustainable diet” OR "sustainable drainage system*" OR "wastewater management" OR "water reuse system*" OR "heat-health action plans" OR "early warning system*" OR "community-based adaptation" OR "climate-resilient healthcare system*" OR "emergency preparedness" OR "renewable energy" OR "solar photovoltaic system*" OR "solar panel*" OR "wind energy" OR "heat pump" OR "low-carbon heating" OR "home insulation" OR "building retrofit" OR "home retrofit" OR "net-zero building*" OR “urban planning” OR "active transport" OR "sustainable transport" OR "active travel" OR "cycling infrastructure" OR "cycle corridor*" OR "cycleway*" OR "greenway*" OR "electric vehicle*" OR "electric bus*" OR "low-emission bus*" OR "sustainable mobility" OR "low emission zone*" OR ULEZ OR "clean air zone*" OR "low-traffic neighbourhood*"). ti,ab,kw. |
|  | 22 | 20 OR 21 |
| Mental health terms | 23 | mental health/ |
|  | 24 | quality of life/ |
|  | 25 | depression/ |
|  | 26 | anxiety/ |
|  | 27 | psychological stress/ |
|  | 28 | mental disorders/ |
|  | 29 | (mental* or psychiatric) adj1 (health or ill* or disord* or disab* or distress* or wellbeing or well-being or resilience).ti,ab,kw. |
|  | 30 | ("subjective wellbeing" or "subjective well-being" or "psychological wellbeing" or "psychological well-being" or “emotional wellbeing” or “emotional well-being”).ti,ab,kw. |
|  | 31 | ((psycho* or mood or trauma or depress* or anxi* or anxious or suicide* or solastalgi* or post-traumatic stress disorder or ptsd or dement*)ab,ti,kw. |
|  | 32 | or/23-31 |
|  | 33 | 19 AND 22 AND 32 |
| Restricting the results to human studies in journal articles in the past ten years | 34 | limit 33 to humans |
|  | 35 | limit 30 to yr="2015 - 2025" |
|  | 36 | limit 31 to journal article |

| **Table S2.** Summary of quality appraisal results using the Mixed-Methods Appraisal Tool (MMAT). Results are grouped by study design as the MMAT uses different criteria for different study designs. Mixed-methods studies were first appraised against the relevant quantitative and qualitative criteria before being appraised against the mixed methods criteria. Responses indicate whether each MMAT criterion was met (Y:Yes), not met (N:No), or not possible to determine (CT:Can’t tell). | | | | | | | | | | | | | | | | |
| --- | --- | --- | --- | --- | --- | --- | --- | --- | --- | --- | --- | --- | --- | --- | --- | --- |
| **RANDOMISED CONTROLLED TRIALS** | | | | | | | | | | | | | | | | |
| Paper | 2.1. Is randomization appropriately performed? | | 2.2. Are the groups comparable at baseline? | | | 2.3. Are there complete outcome data? | | | 2.4. Are outcome assessors blinded to the intervention provided? | | | 2.5 Did the participants adhere to the assigned intervention? | | | Final score | |
| Page et al. 2025 | Y | | Y | | | Y | | | Y | | | Y | | | ***** | |
| **QUANTITATIVE NON-RANDOMISED STUDIES** | | | | | | | | | | | | | | | | |
| Paper | 3.1. Are the participants representative of the target population? | | | 3.2. Are measurements appropriate regarding both the outcome and intervention (or exposure)? | | | 3.3. Are there complete outcome data? | | | 3.4. Are the confounders accounted for in the design and analysis? | | | 3.5. During the study period, is the intervention administered (or exposure occurred) as intended? | | | Final score |
| Ahrentzen et al. 2015 | CT | | | Y | | | Y | | | N | | | Y | | | *** |
| Beshir et al. 2025 | Y | | | Y | | | CT | | | Y | | | Y | | | **** |
| Breysse et al. 2015 | N | | | Y | | | Y | | | Y | | | Y | | | **** |
| Curl et al. 2015 | CT | | | Y | | | Y | | | Y | | | CT | | | *** |
| Grey et al. 2017 | Y | | | Y | | | N | | | Y | | | Y | | | **** |
| Harrison et al. 2025 | CT | | | Y | | | Y | | | CT | | | Y | | | *** |
| Hunter et al. 2021 | Y | | | Y | | | CT | | | Y | | | Y | | | **** |
| Hutchinson et al. 2025 | Y | | | Y | | | CT | | | N | | | Y | | | *** |
| Jalali et al. 2016 | Y | | | Y | | | CT | | | N | | | Y | | | *** |
| Poortinga et al. 2017 | Y | | | Y | | | Y | | | Y | | | N | | | **** |
| Tonn et al. | Y | | | Y | | | N | | | CT | | | Y | | | *** |
| **QUANTITATIVE DESCRIPTIVE STUDIES** | | | | | | | | | | | | | | | | |
| Paper | 4.1. Is the sampling strategy relevant to address the research question? | | | 4.2. Is the sample representative of the target population? | | | 4.3. Are the measurements appropriate? | | | 4.4. Is the risk of nonresponse bias low? | | | 4.5. Is the statistical analysis appropriate to answer the research question? | | |  |
| Panno et al. 2017 | CT | | | CT | | | Y | | | CT | | | Y | | | ** |
| **MIXED METHODS STUDIES** | | | | | | | | | | | | | | | | |
| Paper | Results for the qualitative component (1.1-1.5)* | Results for the quantitative component (4.1 – 4.5) | | | 5.1. Is there an adequate rationale for using a mixed methods design to address the research question? | | | 5.2. Are the different components of the study effectively integrated to answer the research question? | 5.3. Are the outputs of the integration of qualitative and quantitative components adequately interpreted? | | 5.4. Are divergences and inconsistencies between quantitative and qualitative results adequately addressed? | | | 5.5. Do the different components of the study adhere to the quality criteria of each tradition of the methods involved? |  | |
| Grabow et al. 2018 | Y,Y,Y,Y,CT | N,Y,Y,N,Y | | | Y | | | Y | N | | CT | | | Y | *** | |
| Sanz-Mas et al. 2025 | Y,Y,Y,Y,Y | Y,Y,Y,Y,Y | | | Y | | | Y | Y | | Y | | | Y | ***** | |
| Sawyer et al. 2022 | Y,Y,Y,Y,Y | Y,Y,N,N,Y | | | Y | | | Y | Y | | N | | | Y | *** | |
| Walker et al. 2015 | Y,Y,Y,Y,Y | Y,N,Y,N,Y | | | Y | | | Y | Y | | CT | | | N | *** | |

**Criteria for qualitative studies 1.1-1.5: 1.1. Is the qualitative approach appropriate to answer the research question? 1.2. Are the qualitative data collection methods adequate to address the research question? 1.3. Are the findings adequately derived from the data? 1.4. Is the interpretation of results sufficiently substantiated by data? 1.5. Is there coherence between qualitative data sources, collection, analysis and interpretation?*
